# Wastewater tiling amplicon sequencing in sentinel sites reveals longitudinal dynamics of SARS-CoV-2 variants prevalence

**DOI:** 10.1016/j.wroa.2024.100224

**Published:** 2024-04-24

**Authors:** Yu Wang, Gaofeng Ni, Wei Tian, Haofei Wang, Jiaying Li, Phong Thai, Phil M. Choi, Greg Jackson, Shihu Hu, Bicheng Yang, Jianhua Guo

**Affiliations:** a Australian Centre for Water and Environmental Biotechnology, The University of Queensland, St. Lucia, Brisbane, QLD 4072, Australia; b Department of Microbiology, Biomedicine Discovery Institute, Monash University, Melbourne, Victoria, Australia; c MGI Australia Pty Ltd, 300 Herston Road, Herston, Brisbane, QLD 4006, Australia; d Queensland Alliance for Environmental Health Sciences (QAEHS), The University of Queensland, Brisbane, Queensland, Australia; e Water Unit, Health Protection Branch, Queensland Public Health and Scientific Services, Queensland Health, Brisbane, Queensland, Australia

**Keywords:** Wastewater-based epidemiology, Genome sequencing, Variants of concern, Variant dynamics, Disease prevalence

## Abstract

The ongoing evolution of SARS-CoV-2 is a significant concern, especially with the decrease in clinical sequencing efforts, which impedes the ability of public health sectors to prepare for the emergence of new variants and potential COVID-19 outbreaks. Wastewater-based epidemiology (WBE) has been proposed as a surveillance program to detect and monitor the SARS-CoV-2 variants being transmitted in communities. However, research is limited in evaluating the effectiveness of wastewater collection at sentinel sites for monitoring disease prevalence and variant dynamics, especially in terms of inferring the epidemic patterns on a broader scale, such as at the state/province level. This study utilized a multiplexed tiling amplicon-based sequencing (ATOPlex) to track the longitudinal dynamics of variant of concern (VOC) in wastewater collected from municipalities in Queensland, Australia, spanning from 2020 to 2022. We demonstrated that wastewater epidemiology measured by ATOPlex exhibited a strong and consistent correlation with the number of daily confirmed cases. The VOC dynamics observed in wastewater closely aligned with the dynamic profile reported by clinical sequencing. Wastewater sequencing has the potential to provide early warning information for emerging variants. These findings suggest that WBE at sentinel sites, coupled with sensitive sequencing methods, provides a reliable and long-term disease surveillance strategy.

## Highlights

• A tiling amplicon sequencing was used to track the longitudinal dynamics of SARS-CoV-2 variants.
• It can accurately and consistently estimate the disease prevalence trend at the state level.
• The variant dynamics observed in wastewater were largely in line with clinical reports.
• Wastewater sequencing has the potential to provide early warning information for emerging variants.

## Graphical abstract

**Figure:**
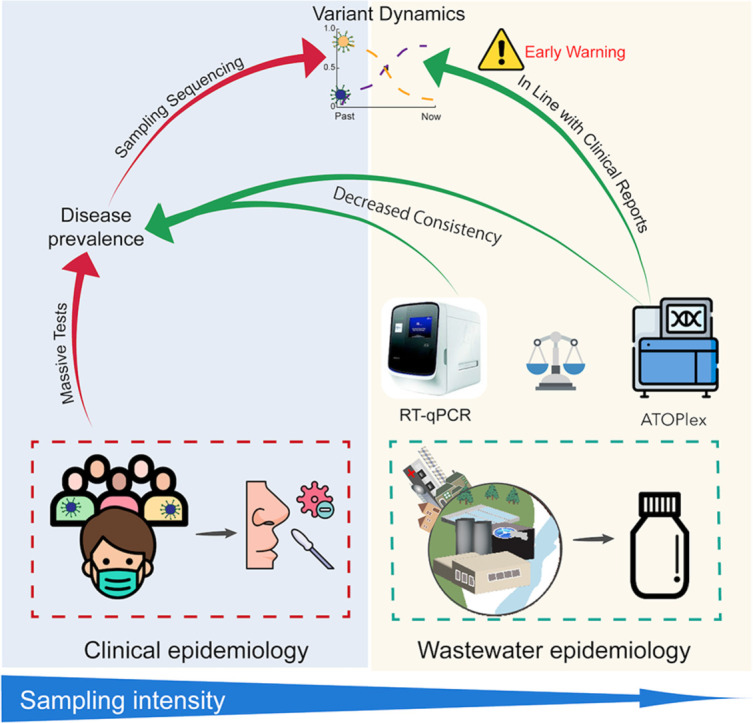

## 1 Introduction

The World Health Organization (WHO) designates evolved variants with genetic mutations associated with increased transmissibility, pathogenicity and immune escape as variants of concern (VOCs). The emergence of SARS-CoV-2 variants with a growth advantage is likely to increase the incidence rate and supplant previous circulating variants (Brito et al., 2022). Since the appearance of the first VOC in 2020, namely Alpha, emergent variants have caused several global surges in COVID-19 (Brito et al., 2022). Therefore, timely identification of SARS-CoV-2 variants and their dynamics is essential for preventing variants spread in the community. Currently, variants monitoring and related genetic characteristics typically rely on clinical sequencing (Levy et al., 2023). However, clinical surveillance is costly, labor-intensive, and inherently prone to sampling bias with grab samples. Additionally, as the global response to COVID-19 strategies evolves, a decreasing number of infections undergo clinical testing, clinical surveillance becoming less effective in revealing variant dynamics (Brito et al., 2022; Karthikeyan et al., 2022). This challenge is exacerbated where medical resources are scarce (Brito et al., 2022; Levy et al., 2023). The risk of new variants emerging calls for sustained monitoring strategies to inform timely preventive measures (Wolfe et al., 2022).

Wastewater-based epidemiology (WBE) enables effective disease surveillance at the community level (Schmitz et al., 2021). The advantage of WBE lies in its ability to offer consistent population coverage, thereby minimizing the sampling bias in clinical testing. It has been adopted as a notional surveillance program worldwide, such as in the US, Australia, and Europe in response to COVID-19 (Ahmed et al., 2020; La Rosa et al., 2020; Li et al., 2022; Sherchan et al., 2020). The application of PCR-based methods has facilitated WBE in monitoring the disease prevalence in communities and providing early warnings of disease outbreaks before clinical surveillance (Corchis-Scott et al., 2021; Medema et al., 2020; Sellers et al., 2022). However, mutations or degradation in N gene, the commonly targeted PCR primer binding region, will reduce the primer binding efficiency. This may lead to false negative PCR results and impeding the timeliness of WBE (Laine et al., 2022; Le et al., 2023). Despite progress in variant-specific PCR assays, identifying multiple variants in wastewater and promptly detecting emerging variants in communities remains a significant challenge (Oloye et al., 2022; Yaniv et al., 2021).

Advanced sequencing retrieves viral genomes from wastewater, enabling the identification of variant-specific mutations (Karthikeyan et al., 2022). This enables distinguishing different variants and gaining insights into their co-occurrence and transition dynamics. However, SARS-CoV-2 RNA in wastewater is present at low concentrations and is prone to fragmentation, posing challenges in obtaining high-quality viral genomes. This complexity further complicates the analysis required for accurate classification of SARS-CoV-2 (Li et al., 2023a). Tiling amplicon sequencing approaches, such as ARTIC, ATOPlex, and Midnight, are demonstrated as sensitive and cost-effective methods for recovering SARS-CoV-2 genomes from wastewater (Freed et al., 2020; Jahn et al., 2022; Wang et al., 2022). By using this technology, wastewater surveillance offers valuable insights into disease prevalence and potential transmission links within communities. The proportion of positive wastewater samples for specific variants correlated with the prevalence of those variants in clinical cases. (Agrawal et al., 2022; Kaya et al., 2022). Moreover, the recovery of high-quality genomes from wastewater uncovers various VOCs introduced across different regions (Bar-Or et al., 2021; Crits-Christoph et al., 2021). Wastewater sequencing at the building level proves effective in the early detection of infection events, enabling rapid responses such as the identification and isolation of positive cases to mitigate potential outbreaks (Karthikeyan et al., 2022). Additionally, WBE surveillance across a university campus has effectively tracked the temporal profile of different variants in a defined region through intensive and frequent sampling (Corchis-Scott et al., 2021; Karthikeyan et al., 2022). However, establishing the intensive WBE surveillance program requires close collaboration between researchers, government, and industry sectors (Sangsanont et al., 2023). This requirement is constrained by limited budgets, particularly as the focus on SARS-CoV-2 decreases. The effectiveness of strategically selected sampling sites in the long-term tracking of disease prevalence over broader regions remains uncertain. Moreover, there has been limited effort in evaluating the utility of wastewater sequencing at sentinel sites for monitoring variant dynamics and contrasting this information with clinical sequencing data.

This study conducted a longitudinal investigation, strategically collecting wastewater samples across three years (from mid-2020 to late-2022) from three municipal sites in Queensland, Australia. The objective was to assess the efficacy of long-term wastewater sequencing surveillance at sentinel sites in estimating disease prevalence and reflecting variant dynamics across large-scale geographical areas. Utilizing ATOPlex sequencing, we successfully tracked the circulating dynamics of SARS-CoV-2 variants and demonstrated its potential for the early detection of emerging variants in wastewater samples. Moreover, ATOPlex has proven to be more reliable than PCR-based methods in estimating disease prevalence trends across various sampling sites. Overall, this study offers valuable insights into the benefits of using WBE at sentinel sites for monitoring disease dynamics in communities.

## 2 Results

### 2.1 ATOPlex enables accurate viral rna quantification and high sensitivity for SARS-CoV-2 identification

The linear regression analysis demonstrated a strong correlation (R^2^*_adj_*=0.99) between normalized sequencing reads and absolute RNA concentration measured by dd-PCR, spanning concentration gradients D1 (6235.58 cp/*μ*L) to D5 (0.63 cp/*μ*L) (Figure S2). Moreover, ATOPlex accurately recovers the SARS-CoV-2 genome, even with mutations present compared to the reference genome (MN908947.3). Overall, ATOPlex's capacity to accurately and sensitively estimate viral concentration and derived genomes enables the qualitative and quantitative of the SARS-CoV-2 virus.

**Fig. 1:**
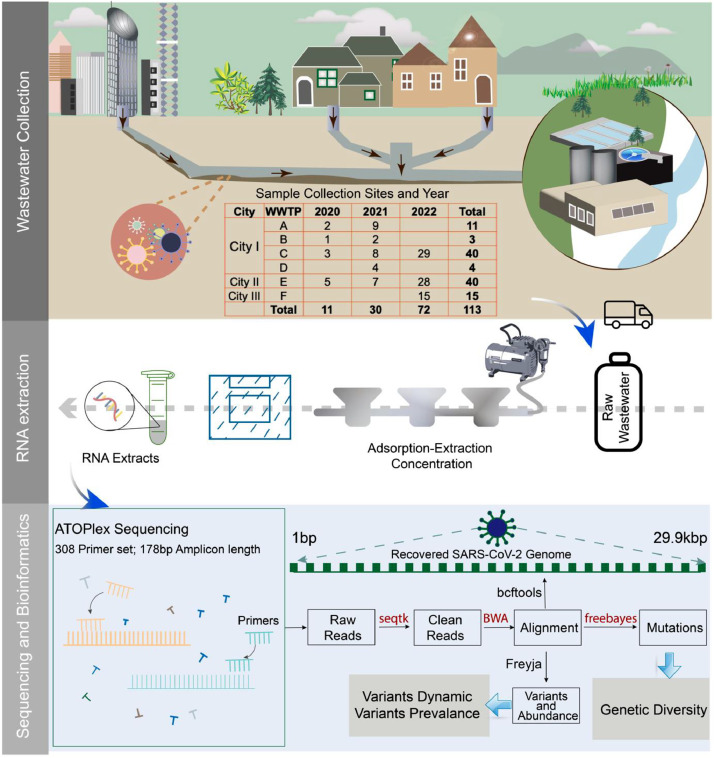
Overview of the wastewater surveillance program. Top panel: Concept of the WBE and collection sites of raw wastewater samples. Middle panel: Wastewater concentration and RNA extraction. Bottom panel: ATOPlex sequencing and bioinformatics analysis.

Of the 113 wastewater samples collected across Queensland (Fig. 1), 109 were classified as positive. The remaining four samples were classified as indeterminate, attributed to low genome coverage (Supplementary S2), which may have resulted from significant degradation and fragmented during long-time storage.

**Fig. 2:**
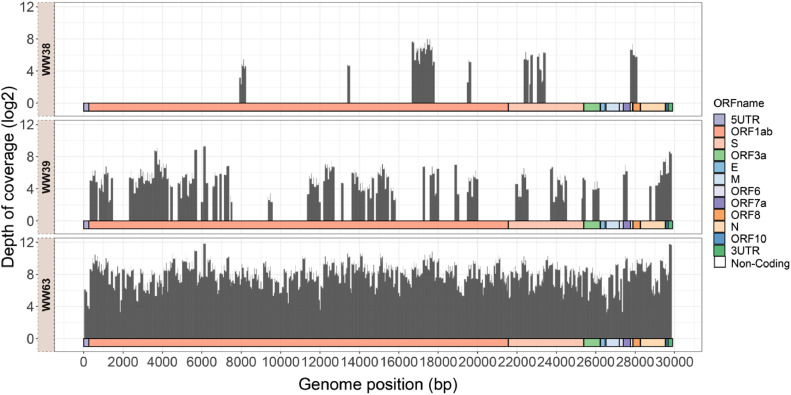
Genome breadth coverage at 10X sequencing depth in wastewater samples. This figure displays the percentage of the reference genome (MN908947.3) covered at a minimum of 10X sequencing depth for ATOPlex positive wastewater samples WW38, WW39, and WW63, with coverage levels of 14.63 %, 50.62 %, and 99.88 %, respectively. Out of the 59 samples achieving over 50 % genome coverage, 49 exhibited coverage higher than 90 %, with the remaining 10 samples ranging between 50 and 90 % coverage. The x-axis indicates the coverage across each nucleotide position of the reference genome, while the y-axis shows the sequencing depth on a log2 scale for each nucleotide position.

To assess the completeness of the recovered viral genome from wastewater, 10X genome coverage (genome breadth coverage, the percentage of genome covered by reads at a sequencing depth of 10 or higher) was visualized (Figure S3). The median genome coverage was 59.70 % (range 0.69–99.88 %), 59 out of 113 wastewater samples exhibited more than 50 % genome coverage. Fig. 2 illustrates three examples of the distribution of sequencing reads with a different genome coverage (10X). Notably, sample WW38 tested negative in the second round RT-qPCR test before sequencing. It is apparent that ATOPlex still can identify the SARS-CoV-2 virus even in instances where the primer regions N1 and N2 commonly used in PCR-based methods were degraded.

### 2.2 Viral load in wastewater reveals temporal disease prevalence trend

**Fig. 3:**
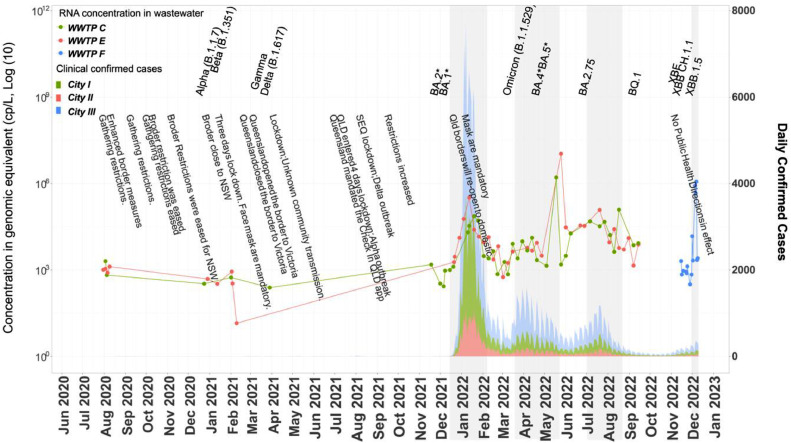
Epidemiological trends of disease prevalence in the study regions. The line graph shows disease trends from wastewater data, and the shaded area reflects daily clinical cases. The grey text shows the public health interventions in Queensland, and black markers note the detection of new variants. The grey background shows the main surges of COVID in Queensland.

The temporal trend of total SARS-CoV-2 RNA concentration in wastewater closely aligns with the confirmed COVID-19 cases (Fig. 3). During 2020 and 2021, Queensland implemented stringent disease control measures to effectively minimise local transmission (Table S2). However, following the opening of the Queensland border on 13 December 2021, there was a marked increase in weekly total cases. The total confirmed cases number is nearly 1.5 times higher in the week after the reopening compared to the week prior (Fig. 3). This increase was mirrored by a corresponding upward trend in total SARS-CoV-2 RNA concentration detected in wastewater (Fig. 3). Additionally, during the study period COVID-19 exhibited four main surges, as captured in both clinical and wastewater surveillance, which are likely associated with the emergence of new variants (Fig. 3). However, a notable lag in the SARS-CoV-2 RNA concentration in wastewater and the peak of clinically confirmed cases during the second surge.

**Fig. 4:**
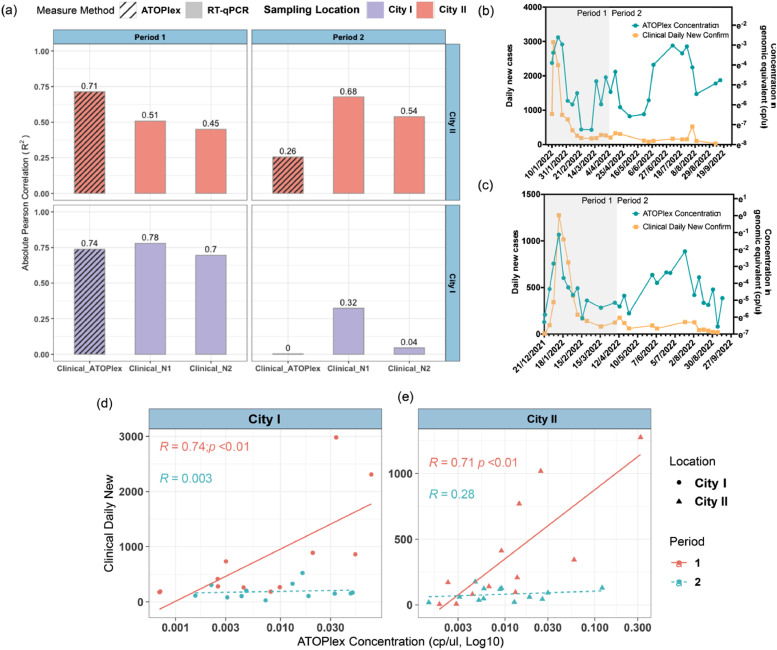
Comparative correlation analysis between RNA concentrations in wastewater and clinically daily confirmed COVID-19 cases reported in corresponding catchment areas. (a) Presents a summary of Pearson correlation coefficients (|R|), depicting the strength of the relationship between the RNA concentrations measured in wastewater and the confirmed cases over the study periods. (b) and (c) Offer a detailed view of the temporal trends in RNA concentration as detected by wastewater sequencing (depicted in blue) compared to the trend in clinically confirmed COVID-19 cases (shown in yellow), highlighting discrepancies and potential underreporting in public health data. (d) and (e) Illustrate the correlation trends between the confirmed cases and RNA concentrations measured by the ATOPlex method across different periods in City I and City II, respectively.

To assess the effectiveness of RT-qPCR and ATOPlex methods in reflecting disease prevalence, we computed the Pearson correlation between total RNA concentrations in wastewater and daily confirmed cases within the catchment area. This analysis was conducted separately for two distinct periods, before and after April 1, 2022, corresponding to shifts in diagnostic approaches. The first period is characterized by the widespread use of clinical PCR tests, while the second represents increased acceptance and adoption of Rapid Antigen Testing (RAT) kits for self-testing. In period 1, ATOPlex showed a stronger and more stable correlation with the confirmed cases in both City I and City II regions. This was followed by a reduced correlation in the subsequent period. Conversely, RT-qPCR which targets N1 or N2 genetic markers, initially exhibited a weaker correlation compared to the ATOPlex method. In the second period, the correlation with RT-qPCR also experienced a decrease in City I but slightly increased in City II. Notably, RT-qPCR maintained a stronger correlation in period 2 compared to ATOPlex (Fig. 4-a). Furthermore, with the reduction in clinical testing from mid-March 2022 onwards, ATOPlex estimated RNA concentration in wastewater consistently surpassed the clinical case counts (Fig. 4-b, c). The comparative correlation analysis in Fig. 4-d, 4e suggests that the estimated disease prevalence in wastewater may be higher than that reported to public health authorities.

Collectively, these results suggest that the disease epidemiological trends in the community can be captured by analysing the viral load in wastewater. Nevertheless, the prevalence estimates derived from wastewater data are increasingly diverging from those obtained through clinical surveillance, particularly with ATOPlex, which displays a more marked divergence.

### 2.3 Early detection and transition patterns of emerging VOCs

**Fig. 5:**
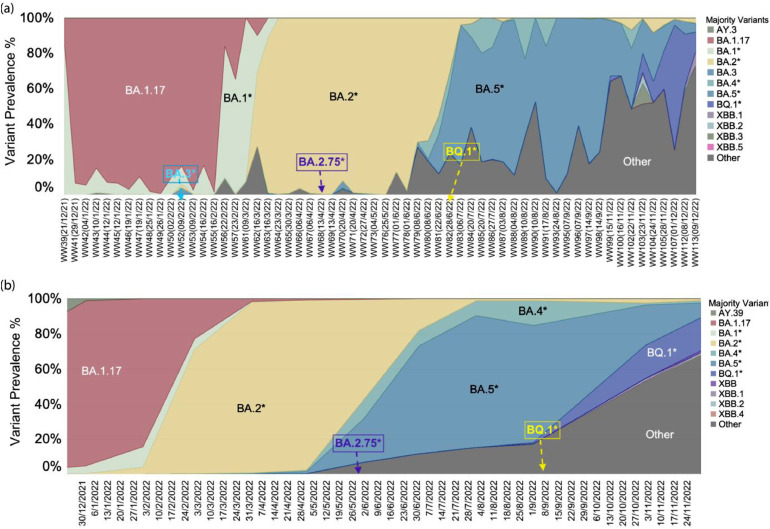
Prevalence of the SARS-CoV-2 variants identified in wastewater and clinical samples during the experimental period is expressed as a percentage. (a) illustrates the prevalence of SARS-CoV-2 variants in 59 sentinel wastewater-derived genomes (10X breadth genome coverage > 50 %). (b) presents the prevalence of variants detected in complete clinical genomes sourced from the GISAID database, corresponding to samples collected on the same day in the whole Queensland state. The details of wastewater and clinical epidemiological data can be found in Supplementary S2.

Intensive clinical monitoring has revealed dynamics of the emerging variants. Considering the limitations of extensive clinical surveillance, we investigated wastewater sequencing at sentinel sites as an alternative strategy to assess the prevalence of circulating variants. Variants detected in wastewater samples showed the main circulating variants were Omicron and its sublineages, with co-occurring variants (e.g. Gamma and Delta) present in low abundance after Queensland border reopened (Fig. 5-a). The prevalence of the BA.2 variant replaced the BA.1.17 variant in mid-March 2022, and then later in July the BA.5* became dominant (Fig. 5-a). These trends align with the variant prevalence observed in clinical sequencing data (Fig. 5-b). For example, clinical data indicated that BA.2 became the predominant circulating variant in mid-March (Fig. 5-b). More importantly, the detection of new variants in wastewater samples could precede their identification in clinical settings (Supplementary S2). In total, 232 variants were detected in both wastewater sequencing and clinical sequencing datasets. Figure S4-a shows the comparison of the first detection date of these variants in each dataset. Given the number of sequenced wastewater samples (accounting for only 0.38 % of the clinical sequenced samples), results show that half of the variants (116) were detected in wastewater samples prior to their detection through clinical surveillance. For instance, signals of the BA.2.75* were found in wastewater on April 13, 2022, preceding its local spread which was reported clinically on July 4, 2022. Similarly, BQ.1* was detected in wastewater in late June, while it was not reported clinically until mid-September. Moreover, the BA.3 was identified in wastewater but not in clinical sequencing, revealing the potential cryptic transmission of these variants in Queensland (Fig. 5-a, b). This finding highlights the potential of wastewater sequencing as an early warning tool enabling effective monitoring of emerging variants that are introduced to the community.

### 2.4 SARS-CoV-2 genetic diversity in wastewater

**Fig. 6:**
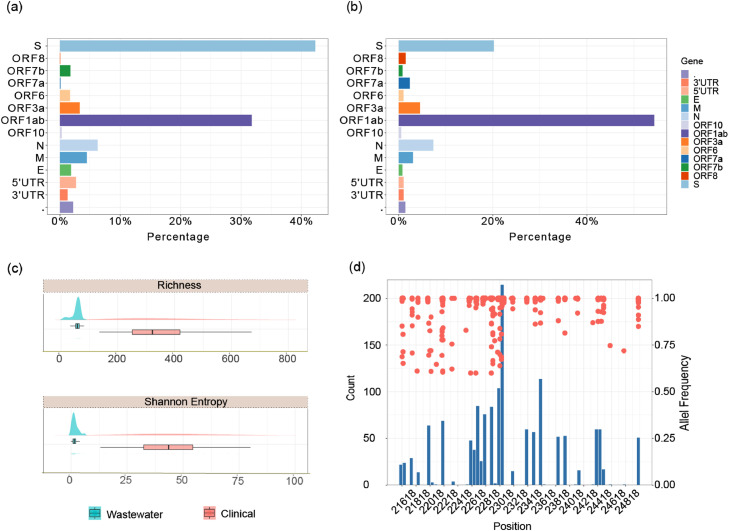
Summary of genetic variations in SARS-CoV-2 genes in wastewater and clinical samples. Pesrtange of mutations that occur on each SARS-CoV-2 gene in wastewater samples (a) and clinical samples (b). Genetic diversity of SARS-CoV-2 in wastewater and clinical samples (c), represented by richness (top) and Shannon Entropy (bottom). Mutations detected in the Spike protein of SARS-CoV-2 in wastewater samples are shown in (d). The histogram (bin width = 50 bp) is the count of the number of each mutation that occurs on the genome position that is aligned with the left y-axis, while the scatter plot represents the allele frequency of each mutation in the corresponding position aligned with the right y-axis.

The identification of SARS-CoV-2 variants heavily relies on the single nucleotide variants (SNVs) associated with each variant, making the genetic information vital for monitoring the emergence of new variants (Sapoval et al., 2023). To determine the potential of wastewater sequencing in detecting the evolution of the virus at the population level, we analyzed the genetic diversity in both wastewater and clinical samples. Overall, the mutation analysis of 59 wastewater samples derived SARS-CoV-2 genomes and 4878 clinical-derived SARS-CoV-2 genomes yielded 3473 and 17,017 mutations, respectively. The ratio of mutations detected in each gene across the SARS-CoV-2 genome is consistent between wastewater samples and clinical samples (Fig. 6-a, b), indicating ATOPlex sequencing reliably recovers the SARS-CoV-2 genome without bias. Genetic diversity metrics of richness and Shannon Entropy suggest the genetic diversity of SARS-CoV-2 in the clinical samples is greater than that in the wastewater samples (Fig. 6-c). Furthermore, Fig. 6-d depicts the 1469 mutations identified in the spike protein within wastewater samples with high frequency. Among these mutations, 1155 are located in the S1 subunit of the spike protein, a critical component where the receptor-binding domain (RBD) is located. This domain directly interacts with the human angiotensin-converting enzyme 2 (ACE2) receptor (Hoffmann et al., 2020). Mutations in this region typically change the virus affinity to human cells, thus posing a high risk of instigating the emergence of novel variants (Hoffmann et al., 2020) (Figure S5). Notably, despite the higher genetic diversity observed in clinical samples, our analysis identified 28 unique spike protein mutations in wastewater samples, whereas they were absent in the clinical counterparts (Supplementary S2).

## 3 Discussion

We show that when wastewater samples tested negative with RT-qPCR, ATOPlex was still able to detect viral signals (Fig. 2). Previous study demonstrated that ATOPlex can detect SARS-CoV-2 RNA at concentrations one order of magnitude lower than the detection limit of RT-qPCR (Ni et al., 2021). Moreover, ATOPlex accurately estimates SARS-CoV-2 RNA concentration and recovers genome when the concentration is as low as 0.63 copies/µL (Figure S2). When fecal sample from individuals testing positive for SARS-CoV-2 enters a WWTP, the viral load in wastewater can reach levels of up to 3 copies/µL (Foladori et al., 2020). These findings support the use of ATOPlex for sensitively estimating RNA concentration in wastewater when there are positive cases in the community.

Our analysis revealed four local outbreaks during the study period through wastewater data, which aligned with the clinical epidemiology (Fig. 3). Additionally, it is expected that total SARS-CoV-2 RNA concentration in wastewater reflects the changes in disease prevalence in the corresponding catchment (Li et al., 2023a). This study observed a robust Pearson correlation between RNA concentrations in wastewater and the number of daily new confirmed cases before April 1, 2022 (Fig. 4). However, in the context of clinical data distortion, an increasing divergence in this correlation was subsequently noted. As clinical reports lose their comprehensive coverage of the population, the divergence between clinical and epidemiological data points to a potential underreporting of new cases. The more sensitive RNA measurements obtained through ATOPlex are likely to provide a more accurate reflection of the actual number of infections within the community. This technology, therefore, plays a crucial role in bridging the gap between reported case numbers and the true prevalence of the virus, when large-scale clinical surveillance is not feasible.

This longitudinal study demonstrates the potential of sentinel wastewater sequencing in tracking the transition and fitness of different variants. The circulating pattern of these variants is aligned with clinical epidemiological reports from Queensland state (Fig. 5-a, b). While intensive clinical sequencing is effective for detecting emerging variants, our analysis underscores the potential of wastewater sequencing as an early warning tool (Figure S4-a). For instance, the BA.4* and BA.5* variants were believed to have contributed to the second COVID-19 surge in Queensland, 44 out of 71 circulating sub-lineages were first detected in wastewater (Fig. 3). Notably, some of these sub-lineages are the dominant circulating lineages, such as BA.5.2.4 and BA.5.6 (Figure S4-b). It should be also mentioned that multiple variants, such as BA.2 and BA.2.33, were detected early through clinical sequencing, underscoring the necessity of complementing wastewater surveillance with clinical surveillance. However, with the decline in clinical sequencing capabilities, wastewater monitoring emerges as a powerful tool for disease surveillance. Additionally, it was noted that the genetic diversity identified in regional wastewater was lower than that observed in the whole-state clinical sequencing data. This could be attributed to the complexity of wastewater samples, where some RNA samples were not of optimal quality at the time of sequencing. Moreover, the sentinel sites in this study did not cover the population from the entire state. Additionally, monitoring mutations that occur in the SARS-CoV-2 genome is vital for identifying and monitoring emerging variants (Agrawal et al., 2022). WBE as a means of monitoring mutations occurring in the SARS-CoV-2 genome is practical where large-scale clinical sequencing is unfeasible. Furthermore, unique mutations with high frequency, reveal the hidden cryptic circulating variants in the community. These mutations could also serve as indicators of emerging variants in the population. However, due to potential mutation noise, these findings require further careful interpretation to better understand the characteristics of SARS-CoV-2.

Although the application of wastewater genomic surveillance is well recognised during the COVID-19 pandemic, several challenges remain. One significant challenge is obtaining high-quality genomes from wastewater samples, which is often hindered by factors such as RNA degradation or low concentrations. ATOPlex sequencing has demonstrated the capacity to recover complete or partial genomes from wastewater samples. However, we observed regions of low sequencing depth in samples collected recently (Figure S3), which underscores the need to refine the ATOPlex primer set. Such optimization is crucial for capturing a high resolution of mutations occurring in the SARS-CoV-2 genome and for the investigation of emerging variants (Agrawal et al., 2022). Moreover, genomes with >50 % coverage for variant analysis are feasible, but we acknowledge the lack of internal or external RNA control in our research represents a limitation. This may impact the accuracy of our RNA quantification and potentially overlook false negative results. The role of controls in verifying RNA recovery rates is indispensable. For future studies to offer more precise comparisons and to diminish the possibility of underestimating viral loads in wastewater, the inclusion of such controls is essential. Additionally, the selection of sampling sentinel sites and sampling frequency should be precise, particularly in areas characterized by high traffic or population density, to ensure a comprehensive understanding of genetic diversity and the variants in circulation. We also observed a delay in reflecting the peak in cases through wastewater surveillance, which could be attributed to the limited sampling size or the changes in clinical report cases to public health sectors (Galani et al., 2022). Future studies should focus on identifying appropriate sampling sites and establishing long-term routine wastewater sequencing monitoring, to support effective public health decision-making and outbreak prevention. In addition, the correlation coefficient (|r|) between RNA concentration in wastewater and clinical parameters (e.g. daily or active total cases) varied in different studies, ranging from 0.14 to 1 (Li et al., 2023b). This might be attributed to various evaluation conditions, such as environmental factors, sampling strategies and epidemiological factors (Li et al., 2023b). Further research is required to assess the potential impact of a variety of factors on the epidemic model, enhancing the accuracy and precision in predicting disease prevalence in community.

## 4 Conclusion

Overall, this longitudinal study demonstrates that ATOPlex sequencing is a reliable method for identifying SARS-CoV-2 in wastewater samples. The sequencing of sentinel wastewater enables the identification of variant introduction and provides insights into their transmission patterns in community. The evolutionary nature of RNA viruses, characterized by constant changes in virulence, poses an evolving threat to public health. As clinical testing declines, WBE can assist public health sectors in understanding variant dynamics. Furthermore, the implementation of WBE as a surveillance tool holds significant promise in enhancing our preparedness and response strategies for other outbreaks and pandemics, not limited to SARS-CoV-2.

## 5 Materials and methods

### 5.1 Study area and sample collection

Wastewater samples were obtained from six sentinel wastewater treatment plants (WWTPs), situated in three different municipalities (namely, City I, II, III) within Queensland, Australia. The sampling sites and the served population of each WWTP included in the study are depicted in Figure S1. All wastewater samples underwent RT-qPCR test to monitor the SARS-CoV-2, in accordance with the local wastewater surveillance program. The detailed RT-qPCR assays were described in supplementary SI and primer/probe information in Table S1 (Li et al., 2023a, 2022) The RT-qPCR results were obtained from Queensland Public Health and Scientific Services (Supplementary S2). The positive samples were earmarked for sequencing to investigate circulating variants. Throughout 2020 and most of 2021, Queensland implemented stringent public health measures, such as tight border restrictions, during which 14 days of quarantine was required to enter the state. As a result, the collected samples exhibited marked low RNA concentrations. Moreover, the RNA in wastewater was subject to degradation over time. Despite the earmarked samples initially testing positive via RT-qPCR, the second round of RT-qPCR results for the samples from 2020 to 2021 turned negative by the time of sequencing in September 2022. In this study, we specifically selected a few samples from 2020/2021 to evaluate the sensitivity of ATOPlex sequencing. Overall, a total of 113 wastewater samples were sent for sequencing, including WWTPs A (*n* = 11), B (*n* = 3), C (*n* = 40), D (*n* = 4), E (*n* = 40), and F (*n* = 15), respectively (Fig. 1). Samples from WWTPs C and E were collected between July 2020 and September 2022, while WWTPs A, B and D ceased collection in 2022. To further investigate the presence of emerging variants that have not been locally endemic in Queensland, 15 grab samples were collected in November-December 2022 from the largest WWTP in Queensland, WWTP F, which serves the busiest locations of Queensland, including the capital city Brisbane and an international airport (Fig. 1).

### 5.2 Wastewater sample preparation, concentration, and RNA extraction

Samples were transported on ice and stored at −20 °C until further analysis. The frozen samples were first thawed at 4 °C followed by wastewater concentration and RNA extraction as described in previous studies (Fig. 1) (Ni et al., 2021; Pastorino et al., 2020). Briefly, 0.5 mL of 25 mM dissolved MgCl_2_ was added to the aliquots of 50 mL raw wastewater samples and incubated at room temperature for 5 min. Samples were then filtered through a 0.45-µm pore-size, 47-mm diameter electronegative HA membrane (Cat No. HAWP04700; Merck Millipore Ltd, Sydney, Australia) using a multichannel filter funnel. All equipment was autoclaved at 121 °C for 20 min prior to use. The membrane was removed from the filtration apparatus, rolled, shredded and placed into a 2 ml beat tube from RNeasy PowerMicrobiome Kit (Cat No. 26,000–50; Qiagen, Hilden, Germany) using two sterile tweezers. RNA was then extracted following the manufacturer's instructions with the following modifications: (1) 100 μL of phenol–chloroform–isoamyl alcohol (25:24:1) was added before the lysis step to protect RNA integrity; (2) Beat-beating for 60 s with Minilys® homogenizer at medium speed; (3) IRS solution was increased to 200 μL to remove more inhibitors, and (4) RNA eluted with 50 μL Nuclease free water (Biolab) and pass-through spin column twice to ensure maximum RNA yield. RNA extracts were stored at −80 °C before sequencing.

### 5.3 Positive control preparation

A commercially available RNA positive control was used to prepare a serial dilution with 5 orders of gradient (D1-D5), as shown in Figure S1 (b). Briefly, 10 *μ*L RNA was reverse transcribed to cDNA using the high-capacity cDNA reverse transcription kit (Cat No. 4,368,814, Applied Biosystems™, CA, USA) and serially diluted by 5 orders of magnitude with nuclease-free water (Bar-Or et al., 2021). The concentration of each positive control gradient was measured by droplet digital Polymerase Chain Reaction (dd-PCR) assay, as described in a previous study (Ni et al., 2021), with the concentration ranging from 6235.58 to 0.63 copies/*μ*L.

### 5.4 ATOPlex sequencing

A total of 113 wastewater RNA extracts and 5 positive control gradients (in triplicate) were sequenced using the ATOPlex SARS-CoV-2 full-length genome panel which included 308 primer pairs (MGI, China) as described previously. Briefly, sample RNA was reverse transcribed to cDNA with random hexamers (5′-NNNNNN-3′). Then, 200 copies of Lambda phage DNA were dosed into each sample as an internal control to set a baseline for result quantification. The resulting product undergoes two steps of PCR amplification followed by purification, with the first PCR of 13 cycles for target enrichment, and the second PCR of 27 cycles for barcoding and product amplification. The process was completed using the automation sample processing system MGISP-960. The purified final PCR products were quantified using Qubit Quant-iT™ dsDNA Assay Kits (Cat No. Q33120, ThermoFisher, CA, USA). Subsequently, PCR products of 96 samples were pooled into two pools, with each pool containing 48 samples and 400 ng of library. The pooled dsDNA libraries were denatured to generate linear ssDNA, circularized using a splint oligo that is complementary to the start and end of linear ssDNA, followed by digestion reaction and purification to remove non-circularized DNA debris. The process was completed manually using the MGIEasy Dual Barcode Circularization Kit (MGI, China) to produce purified single-strand circularized products. The ssDNA circularized product was then used as a template to generate DNA nanoballs (DNBs) by performing a rolling circle amplification reaction. The DNBs were introduced into flow cells containing patterned arrays of DNB binding sites for sequencing. The sequencing was done on a DNBSeq-G400 sequencer running for 24 h to get 8 M reads for each sample at the MGI Australia Demo Lab.

### 5.5 Data analysis and interpretation

The sequencing raw data were analysed with the bioinformatic pipeline for multiplex-PCR data developed by MGI (MGI, 2022). The established pipeline is used to analyse raw sequencing data, including genome alignment, mutation calling, consensus genome generation and identification (Fig. 1). ATOPlex categorizes wastewater samples as positive, negative, or indeterminate. This classification is based on normalizing the SARS-CoV-2 reads to the spike-in lambda phage DNA control reads and evaluating genome coverage, as outlined in previous studies (Ahmed et al., 2022; Ni et al., 2021). To examine the relationship between RNA concentration and ATOPlex read numbers, a regression analysis was conducted. This analysis utilized data from RNA concentrations measured by ddPCR and normalized reads from ATOPlex sequencing of the positive control dilution series D1-D5, as previously described (Ni et al., 2021). Pearson correlation was then employed to assess the correlation between the RNA concentration in wastewater with the daily new confirmed cases across to periods, before and after 1st April 2022. Following the lifting of domestic border restrictions and the emergence of the Omicron variant, there was a shortage of RAT kits and an increase in prices. Thus, by April 2022, PCR testing remained the preferred testing method, with the positive RAT cases required to be reported to the public health sector as suggested by government public health directions (COVID-19 National Incident Room Surveillance Team, 2022a). During this period, the PCR testing rate gradually decreased while the public acceptance of RAT kits increased. Specifically, from mid-January to mid-April 2022, the number of PCR tests administered per 1000 individuals per week in Queensland dropped from over 50 to approximately 15 (COVID-19 National Incident Room Surveillance Team, 2022b). The COVID-19 daily new confirmed case data in the study areas were sourced from the Queensland Government Open Data Portal (https://www.data.qld.gov.au).

The higher coverage and depth underpin a greater likelihood of accurate sequencing and mutation detection at that position (Agrawal et al., 2022). To ensure data precision, only samples identified as positive and genomes with a coverage at least 50 % (genome breadth coverage, the percentage of genome covered by reads at 10X) were included for variant identification and investigation of genetic diversity. Freyja (version 1.3.11), a well-established bioinformatics pipeline, was utilized to identify co-circulating variants and their prevalence in wastewater samples (Karthikeyan et al., 2022). It has proven effective in detecting variants from genomes derived from wastewater, even when genomic coverage is as low as 50 % (Barnes et al., 2023). Clinical genomic data from Queensland were sourced from the GISAID database (Khare et al., 2021). The clinical genomes were then grouped by collection date, and mutations in the groups were obtained using snp-sites V2.5.1 (Page et al., 2016) the mutation frequency was calculated using a custom script. Genetic diversity of the wastewater samples and clinical samples was measured by the richness (count of the number of mutations present in the sample) and Shannon Entropy (H(x) = - Σ p(x) log₂(p(x), where p(x) is the allele frequency at the position x), the distribution of each mutation and degree of genetic diversity in the sample).

## CRediT authorship contribution statement

**Yu Wang:** Writing – original draft, Visualization, Methodology, Formal analysis, Data curation. **Gaofeng Ni:** Writing – review & editing, Supervision, Methodology, Conceptualization. **Wei Tian:** Methodology, Formal analysis. **Haofei Wang:** Methodology. **Jiaying Li:** Writing – review & editing, Resources. **Phong Thai:** Writing – review & editing, Resources. **Phil M. Choi:** Writing – review & editing, Resources, Data curation. **Greg Jackson:** Writing – review & editing, Resources. **Shihu Hu:** Writing – review & editing, Supervision, Methodology, Conceptualization. **Bicheng Yang:** Methodology. **Jianhua Guo:** Writing – review & editing, Supervision, Funding acquisition, Conceptualization.

## Declaration of competing interest

The authors declare that they have no known competing financial interests or personal relationships that could have appeared to influence the work reported in this paper.

## Data Availability

All the raw sequencing data are available at the National Center for Biotechnology Information (NCBI) Sequence Read Archive (SRA) under BioProject number PRJNA945486.

## Appendix Supplementary materials

Image, application 1

Image, application 2
