# Supplementary material for: Wastewater tiling amplicon sequencing in sentinel sites reveals longitudinal dynamics of SARS-CoV-2 variants prevalence
Source: Water Res X. 2024 Apr 24;23:100224. doi: 10.1016/j.wroa.2024.100224 (PMC11070618; doi:10.1016/j.wroa.2024.100224)
Supplement: Supplementary file 1 [file mmc1.docx]

**Supporting Information**

**Wastewater Tiling Amplicon Sequencing in Sentinel Sites Reveals Longitudinal Dynamics of SARS-CoV-2 Variants Prevalence**

Yu Wang^1^, Gaofeng Ni^2*^, Wei Tian^3^, Haofei Wang^3^, Jiaying Li^4^, Phong Thai^4^, Phil M. Choi^5^, Greg Jackson^5^, Shihu Hu^1^, Bicheng Yang^3^, Jianhua Guo^1*^

^1^Australian Centre for Water and Environmental Biotechnology, The University of Queensland, St. Lucia, Brisbane, QLD 4072, Australia

^2^Department of Microbiology, Biomedicine Discovery Institute, Monash University, Melbourne, Victoria, Australia

^3^MGI Australia Pty Ltd, 300 Herston Road, Herston, Brisbane, QLD 4006, Australia

^4^Queensland Alliance for Environmental Health Sciences (QAEHS), The University of Queensland, Brisbane, Queensland, Australia

^5^Water Unit, Health Protection Branch, Queensland Public Health and Scientific Services, Queensland Health, Brisbane, Queensland, Australia

*Corresponding author:

Jianhua Guo, Email: [jianhua.guo@uq.edu.au](mailto:jianhua.guo@uq.edu.au)

Gaofeng Ni, Email: [gaofeng.ni@monash.edu](mailto:gaofeng.ni@monash.edu)

This file includes:

Figure S1. Wastewater sampling sites and positive control preparation.

Figure S2. ATOPlex sequencing for SARS-CoV-2 positive control samples.

Figure S3. Sequencing depth of the wastewater samples.

Figure S4. Comparative timeline of first detection for SARS-CoV-2 variants via wastewater and clinical sequencing.

Figure S5. Visualization of the S gene (MN908947.3:21,563~25,384) and the function of each structure.

Table S1. Primers, probes and cycling parameters are used in the RT-qPCR method.

Table S2. Key timeline of COVID-19-related events and public health response activities announced by the Queensland government.

**RT-qPCR measurements for real wastewater samples**

The RT-qPCR test that targeting the N genes (CDC N1, CDC N2) of the SARS-CoV-2 genome, was employed for detecting SARS-CoV-2 RNA in wastewater samples. Samples exhibiting a cycle threshold (Ct) growth curve below 40 cycles (< 40 Ct) were classified as positive (Ahmed et al., 2021; CDC, 2019; Li et al., 2022). Specifically, the PCR mixture included US CDC N1/N2 10 *μL* Supermix, 2019-nCoV Kit (500 nM of forward primer, 500 nM of reverse primer and 125 nM of probe) (Catalogue No. 10006606), and 0.4 *μL* of iScript reverse transcriptase and 3 *μL* of template RNA. All RT-qPCR reactions were performed in triplicate and the mean Ct value for each sample can be found in supplementary S2.


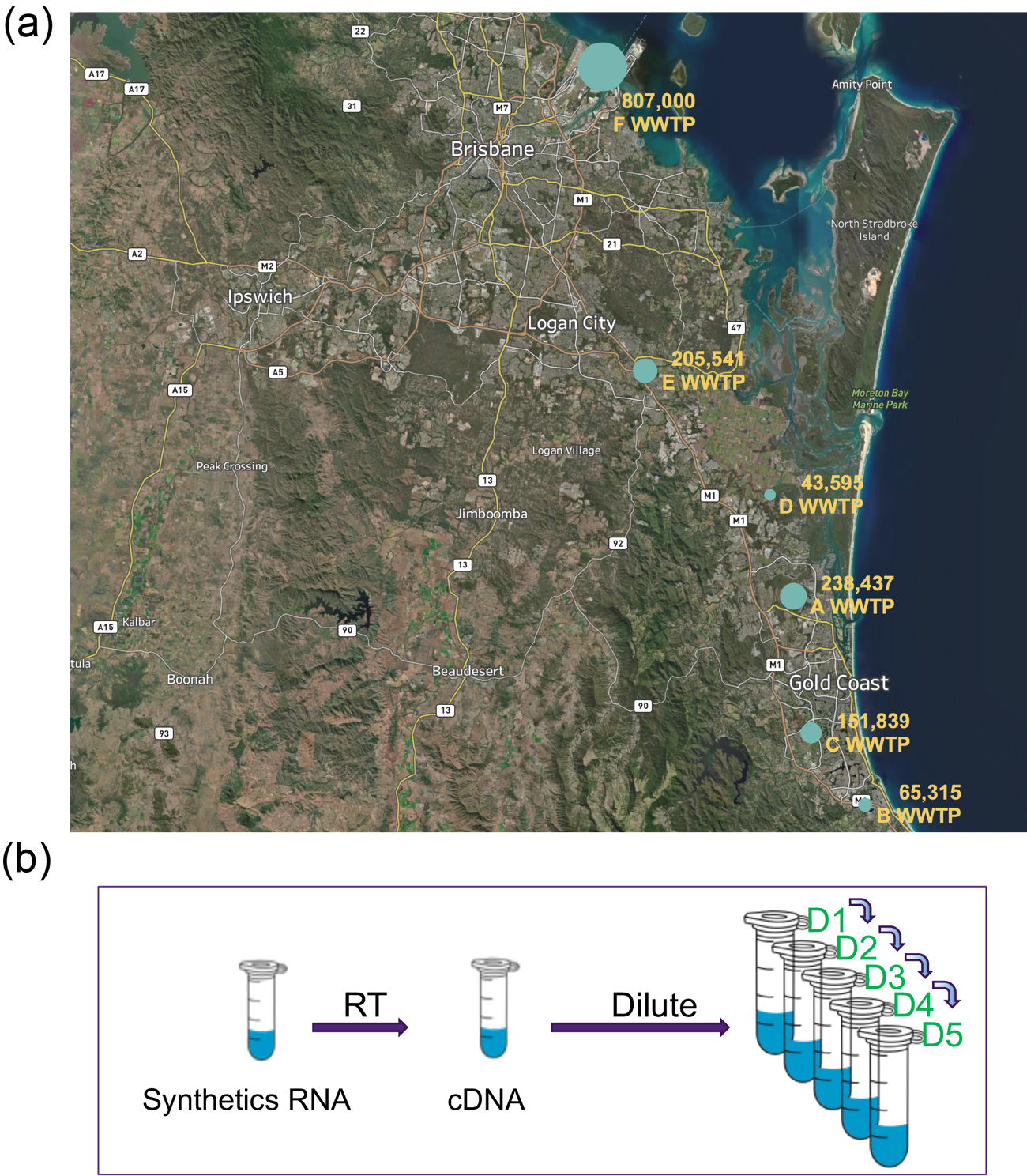


Figure S1. Wastewater sampling sites and positive control preparation. (a) Map of the sampling WWTPs. The dots on the map are the locations of the WWTPs and the number labelled below is the population served by the WWTP. (b) Positive control dilution series for sequencing.


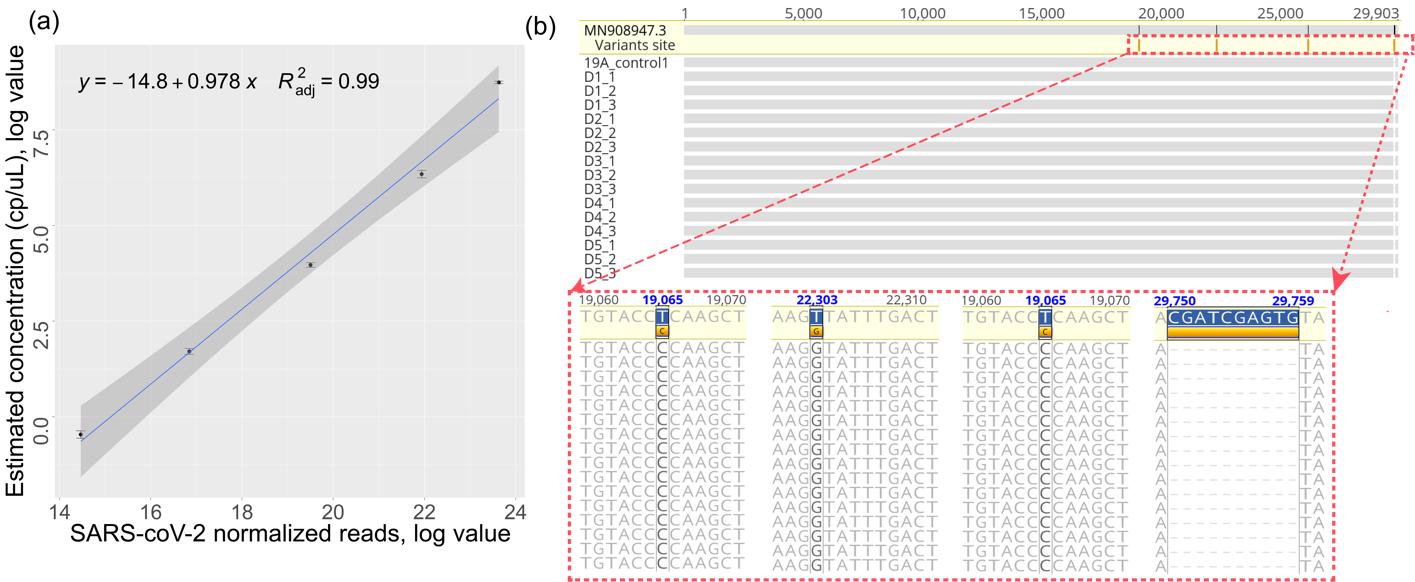


Figure S2. ATOPlex sequencing for SARS-CoV-2 positive control samples. (a) Linear relationship between normalized ATOPlex SARS-CoV-2 reads and viral concentration measured by dd-PCR at log value. (b) Sequence alignments of the reference genome (MN908947.3) and ATOPlex recovered genome across serial dilution D1-D5 align to the positive control 19A genome. Nucleotides that differ from the positive control genome are highlighted in black and have detailed alignment information enlarged.


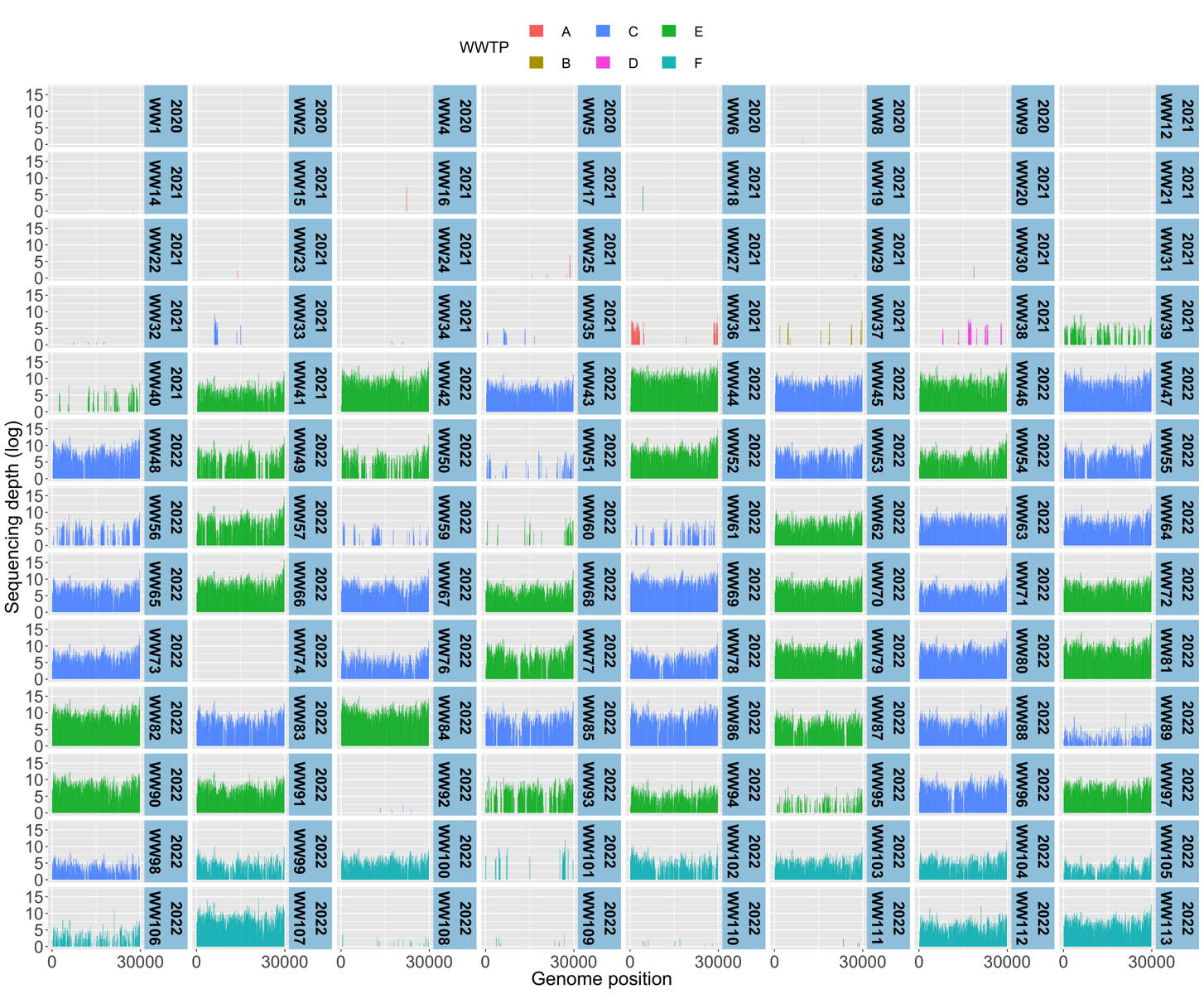


Figure S3 Sequencing Depth of the wastewater samples. The x-axis represents the nucleotide positions in respect to the reference genome (MN908947.3) and the y-axis represents the sequencing depth at each position.


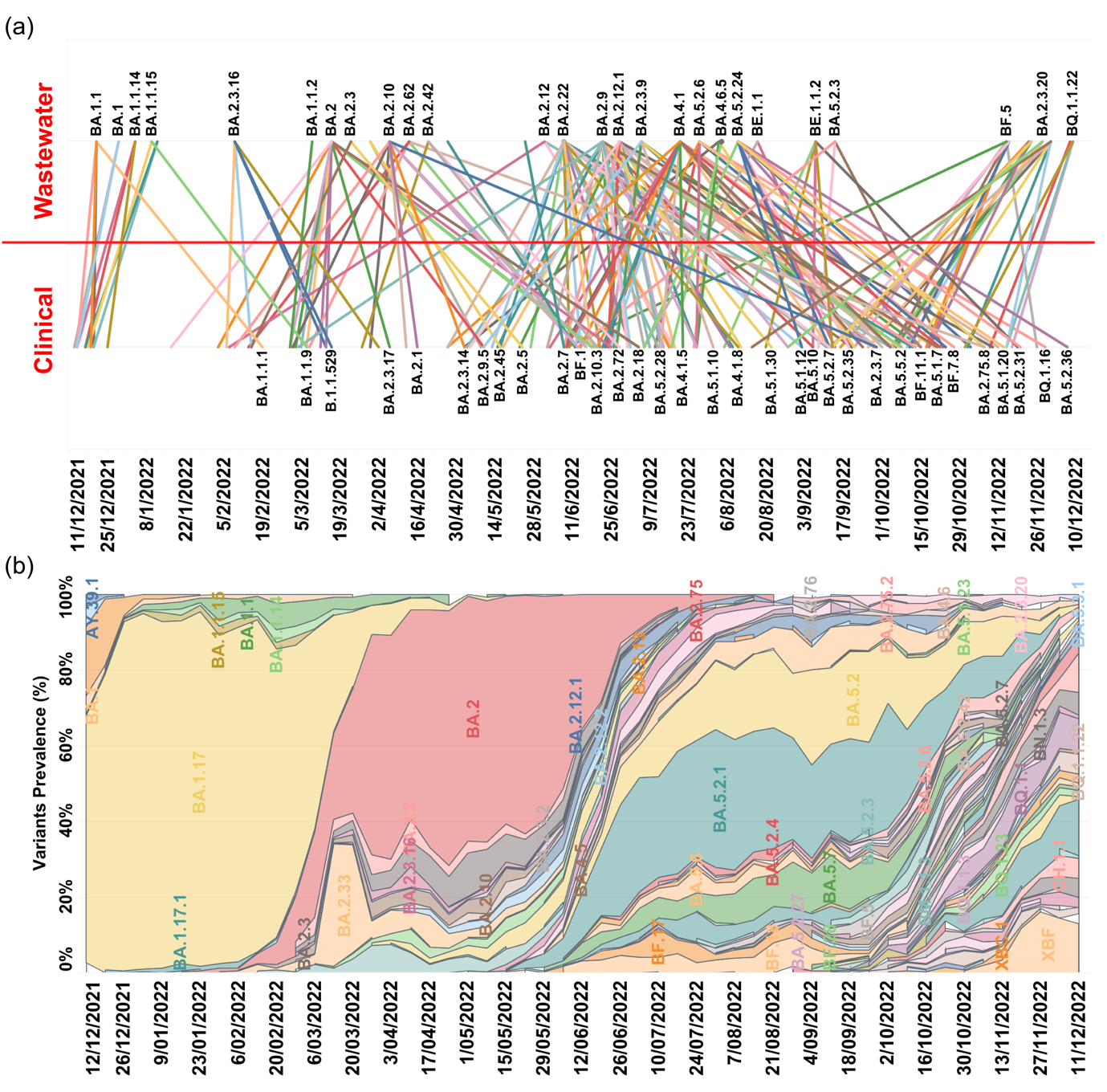


Figure S4. Comparative Timeline of First Detection for SARS-CoV-2 Variants via Wastewater and Clinical Sequencing. (a) This figure illustrates the chronological emergence of various SARS-CoV-2 variants, identified through wastewater sequencing (displayed in the upper part) and clinical sequencing (indicated in the lower part) datasets. The same variants in two datasets were linked by lines. (b) The prevalence of SARS-CoV-2 variants detected via clinical sequencing in Queensland. Due to space limitations, not all variant labels could be displayed. The colour scheme aids in distinguishing between variants without representing their specific identities.


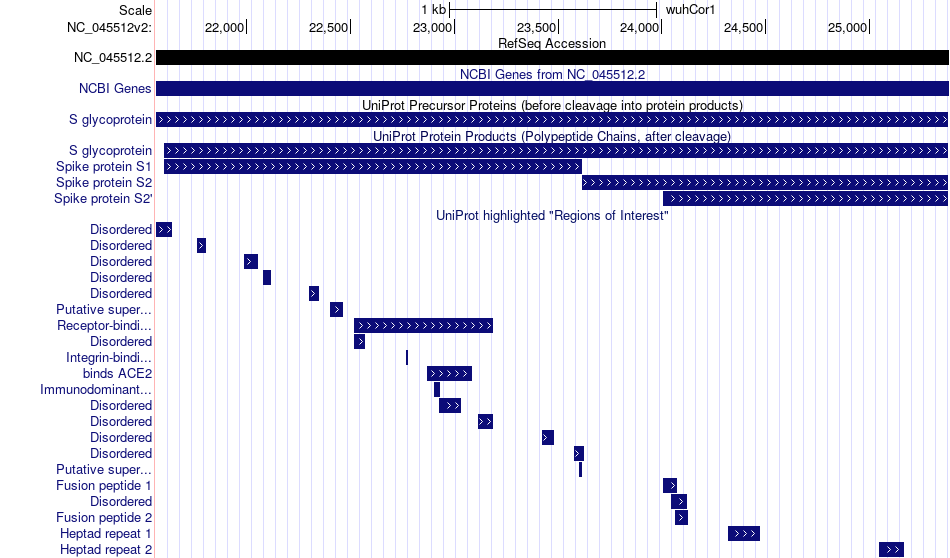


Figure S5 Visualization of the S gene (MN908947.3:21,563~25,384) and the function of each structure. (Fernandes et al., 2020)

Primers, probes and cycling parameters are used in the RT-qPCR method.

| **Organisms** | **Target gene** | **Assay name** | **Sequence (5’–3’)** | **Cycling parameters** |
| --- | --- | --- | --- | --- |
| SARS-CoV-2 | N protein | CDC N1 | F-GACCCCAAAATCAGCGAAAT  R-TCTGGTTACTGCCAGTTGAATCTG  P-FAM- ACCCCGCATTACGTTTGGTGGACC-BHQ1 | 50°C for 10 min for RT; 95°C for 5 min and 45 cycles of 95°C for 10 s, 55°C for 30 s. |
|  |  | CDC N2 | F-TTACAAACATTGGCCGCAAA  R-GCGCGACATTCCGAAGAA  P-FAM- ACAATTTGCCCCCAGCGCTTCAG-BHQ1 | 50°C for 10 min for RT; 95°C for 5 min and 45 cycles of 95°C for 10 s, 55°C for 30 s. |

Table S2 Key timeline of COVID-19-related events and public health response activities announced by the Queensland government.

| **Date** | **Event** | **Note** |
| --- | --- | --- |
| 30/1/20 | WHO declare Public health emergency of international concern. |  |
| 1/2/20 | Australia implemented the travel restrictions of China. |  |
| 11/2/20 | WHO announced the name for the new coronavirus disease: COVID-19. |  |
| 11/3/20 | WHO characteristic COVID -19 as pandemic. |  |
| 20/3/20 | Travel bans on foreign nationals entering Australia. |  |
| 21/3/20 | QLD border close for non-essential traveller. | Broder close |
| 23/3/20 | Social distance applied in QLD. |  |
| 25/3/20 | Australian citizens and Australian permanent residents are restricted from travelling overseas. |  |
| 28/3/20 | All people entering Australia are required to undertake a mandatory 14-day quarantine at designated facilities (e.g., hotels) in their port of arrival. |  |
| 29/3/20 | Both indoor and outdoor public gatherings limited to two persons only. |  |
| 16/5/20 | Gathering restrictions were eased. |  |
| 1/7/20 | QLD border reopened expect to Victoria. | Broder open |
| 24/7/20 | Gathering restrictions implemented. |  |
| 2/8/20 | QLD enhanced border measures. | Broder restriction. |
| 2/9/20 | Gathering restrictions implemented. |  |
| 25/9/20 | Gathering restrictions eased. |  |
| 1/10/20 | Gathering restrictions further eased. Queensland border zone no longer exists, New South Wales border zone extended. | Broder restriction was eased. |
| 1/11/20 | Restrictions were eased for NSW. |  |
| 21/12/20 | Broder close to NSW. | Broder close. |
| 8/1/21 | Greater Brisbane enters a three-day lock down. Face masks are mandatory. | Lockdown to 11/1/21; B.1.1.7 (Alpha) |
| 13/2/21 | Queensland closed the border to Victoria. | Broder close |
| 27/2/21 | Queensland opened the border to Victoria. | Broder open |
| 29/3/21 | Brisbane enters three days lock down. Queensland entered a period of higher level restrictions for Greater Brisbane. | Lockdown to 01/04/2021; Unknown community transmission. |
| 19/6/21 | Queensland announced all travellers from anywhere in Australia or New Zealand must complete a Queensland Travel Declaration. |  |
| 25/6/21 | Queensland mandated the Check in QLD app. |  |
| 29/6/21 | QLD announced a 3-day lockdown and extend 24h. | Lockdown to 03/07/2021; Alpha outbreak. |
| 1/8/21 | SEQ lockdown. | Lockdown to 08/08/2021; Delta outbreak. |
| 11/9/21 | Restrictions increased. |  |
| 13/12/21 | Qld borders re-open to domestic. | Broder reopen to all states; vaccine rate over 92.5% |
| 1/1/22 | Masks are still mandatory. |  |
| 31/10/22 | No Public Health Directions in effect. |  |

**Reference**

Ahmed, W. et al., 2021. SARS-CoV-2 RNA monitoring in wastewater as a potential early warning system for COVID-19 transmission in the community: A temporal case study. Sci. Total Environ. 761, 144216. https://doi.org/10.1016/j.scitotenv.2020.144216

CDC, F., 2019. nCoV real-time RT-PCR diagnostic panel. Fact Sheet Healthc. Provid.

Fernandes, J.D. et al., 2020. The UCSC SARS-CoV-2 Genome Browser. Nat. Genet. 52, 991–998. https://doi.org/10.1038/s41588-020-0700-8

Li, J. et al., 2022. Monitoring of SARS-CoV-2 in sewersheds with low COVID-19 cases using a passive sampling technique. Water Res. 218, 118481. https://doi.org/10.1016/j.watres.2022.118481
